# Preclinical Rationale for Targeting the PD-1/PD-L1 Axis in Combination with a CD38 Antibody in Multiple Myeloma and Other CD38-Positive Malignancies

**DOI:** 10.3390/cancers12123713

**Published:** 2020-12-10

**Authors:** Christie P. M. Verkleij, Amy Jhatakia, Marloes E. C. Broekmans, Kristine A. Frerichs, Sonja Zweegman, Tuna Mutis, Natalie A. Bezman, Niels W. C. J. van de Donk

**Affiliations:** 1 Department of Hematology, Cancer Center Amsterdam, Amsterdam University Medical Center, Vrije Universiteit Amsterdam, 1081 HV Amsterdam, The Netherlands; c.verkleij@amsterdamumc.nl (C.P.M.V.); m.broekmans@amsterdamumc.nl (M.E.C.B.); k.frerichs@amsterdamumc.nl (K.A.F.); s.zweegman@amsterdamumc.nl (S.Z.); t.mutis@amsterdamumc.nl (T.M.); 2 Bristol-Myers Squibb, Redwood City, CA 94063, USA; amy.jhatakia@bms.com (A.J.); nbezman@gmail.com (N.A.B.); 3 Arsenal Bio, San Francisco, CA 94080, USA

**Keywords:** immunotherapy, multiple myeloma, tumor microenvironment, PD-1, PD-L1, CD38, daratumumab, checkpoint inhibitor, nivolumab

## Abstract

**Simple Summary:**

The CD38-targeting antibody daratumumab mediates its anti-myeloma activities not only through direct effects on tumor cells, but also by its effects on T-cell immunity through depletion of CD38^+^ immune suppressor cells. We hypothesized that combining daratumumab with modulators of other potent immune inhibitory pathways, such as the PD-1/PD-L1 axis, may further improve its efficacy. We show that during MM progression there is increased expression of the PD-1/PD-L1 pathway components in the bone marrow microenvironment. Although nivolumab (a PD-1 checkpoint inhibitor) moderately increased T-cell frequencies in ex vivo experiments with bone marrow samples from MM patients, no single agent activity was observed, and addition of nivolumab did not enhance the activity of daratumumab in these short-term assays. However, with a longer treatment duration, in mouse experiments, we demonstrate that anti-CD38 and anti-PD-1 antibodies synergize to eradicate MM cells. In addition, our results suggest that this combined immunotherapeutic approach may also be beneficial in other CD38-positive malignancies.

**Abstract:**

The CD38-targeting antibody daratumumab mediates its anti-myeloma activities not only through Fc-receptor-dependent effector mechanisms, but also by its effects on T-cell immunity through depletion of CD38^+^ regulatory T-cells, regulatory B-cells, and myeloid-derived suppressor cells. Therefore, combining daratumumab with modulators of other potent immune inhibitory pathways, such as the PD-1/PD-L1 axis, may further improve its efficacy. We show that multiple myeloma (MM) cells from relapsed/refractory patients have increased expression of PD-L1, compared to newly diagnosed patients. Furthermore, PD-1 is upregulated on T-cells from both newly diagnosed and relapsed/refractory MM patients, compared to healthy controls. In short-term experiments with bone marrow samples from MM patients, daratumumab-mediated lysis was mainly associated with the MM cells’ CD38 expression levels and the effector (NK-cells/monocytes/T-cells)-to-target ratio, but not with the PD-L1 expression levels or PD-1^+^ T-cell frequencies. Although PD-1 blockade with nivolumab did not affect MM cell viability or enhanced daratumumab-mediated lysis in short-term ex vivo experiments, nivolumab resulted in a mild but clear increase in T-cell numbers. Moreover, with a longer treatment duration, PD-1 blockade markedly improved anti-CD38 antibody-mediated cytotoxicity in vivo in murine CD38^+^ tumor models. In conclusion, dual targeting of CD38 and PD-1 may represent a promising strategy for treating MM and other CD38-positive malignancies.

## 1. Introduction

Daratumumab, a first in class human monoclonal antibody targeting CD38, is active and well tolerated as a single agent [1,2] and in combination with standards-of-care in both newly diagnosed [3,4,5] and relapsed/refractory multiple myeloma (MM) patients [6,7,8]. Daratumumab has several modes of action, including direct on-tumor effects, such as complement-dependent cytotoxicity (CDC), antibody-dependent cellular cytotoxicity (ADCC), and antibody-dependent cellular phagocytosis (ADCP) [9,10,11]. In addition, daratumumab eliminates CD38^+^ immune suppressor cells, such as regulatory T-cells (Tregs), regulatory B-cells, and myeloid-derived suppressor cells (MDSCs), resulting in induction of T-cell expansion and enhancement of the cytotoxic activity of T-cells [12,13,14,15,16,17]. However, not all patients respond to single-agent daratumumab or daratumumab-based combination therapy [14]. Furthermore, the majority of patients who initially respond to daratumumab-based therapy eventually develops progressive disease [14]. Novel, rationally designed combination therapies, based on mechanism of action, may contribute to a further improvement in the outcome of MM patients treated with daratumumab.

Programmed cell death-ligand 1 (PD-L1) delivers an inhibitory signal through the immune checkpoint programmed cell death-protein 1 (PD-1) on T-cells, and thereby impairs the host-anti-tumor immune response in several types of cancer. Blockade of PD-1 or PD-L1 improves T-cell-mediated killing of tumor cells, and antibodies targeting PD-1 and PD-L1 have shown marked clinical activity in solid tumors [18,19], as well as in hematologic malignancies such as Hodgkin lymphoma [20]. MM patients frequently express high levels of PD-L1 and PD-1 on their tumor cells and T-cells, respectively. In vitro studies showed that PD-L1 expressing MM cells are protected against MM-specific T-cells, which could be reversed by anti-PD-1 or PD-L1 antibodies [21,22,23]. Furthermore, blocking PD-1 improved the T-cell responses to an autologous dendritic cell (DC)/MM fusion vaccine ex vivo [24]. In addition, blockade of the PD-1/PD-L1 axis alone, or in combination with irradiation or vaccine administration, improved survival in MM mouse models [25,26,27,28]. As a single agent, the anti-PD-1 antibody, nivolumab, induced durable stable disease in 67% of RRMM patients, but no objective responses were observed [29]. Immune modulation through targeting CD38, combined with blockade of the PD-1/PD-L1 axis, may lead to improved T-cell activity and therefore better anti-MM efficacy.

Here we aimed to increase our understanding of cell surface expression of CD38, PD-L1, and PD-1 in a large number of MM patients in different stages of the disease, including those with heavily pretreated, daratumumab-refractory disease. Moreover, we studied the impact of the PD-L1 and PD-1 expression levels on the ability of daratumumab to induce MM cell lysis in short-term ex vivo cytotoxicity assays. We further assessed the anti-tumor immune response following treatment with anti-mouse CD38 as a single agent or in combination with an anti-PD-1 antibody in immunocompetent mouse tumor models. We demonstrate the immunomodulatory effects of an anti-CD38 antibody in vivo (a decrease in immune suppressor cells) and show that targeting two pathways, CD38 and PD-1, can result in enhanced anti-tumor effects vs. either pathway alone. Together, these data highlight the potential of targeting CD38 and PD1/PD-L1 pathways as a promising treatment strategy for CD38-positive malignancies.

## 2. Results

### 2.1. Expression of PD-L1 and CD38 on Tumor Cells and Frequency of PD-1^+^ T-Cells in MM and Primary Plasma Cell Leukemia Patients

#### 2.1.1. PD-1/PD-L1 Axis and CD38 Expression in MM Patients

We first investigated the expression of PD-L1 on tumor cells in bone marrow (BM) samples from a large number of patients with newly diagnosed (ND) MM (*n* = 37), daratumumab-naïve relapsed/refractory (RR) MM (*n* = 43; median of 3 prior lines of therapy), and daratumumab-refractory MM (*n* = 41; median of 6 prior lines of therapy). These BM samples were analyzed by flow cytometry, whereby normal BM samples obtained from healthy donors with a comparable age as the MM patients (*n* = 11) were used as the control.

**Figure 1:**
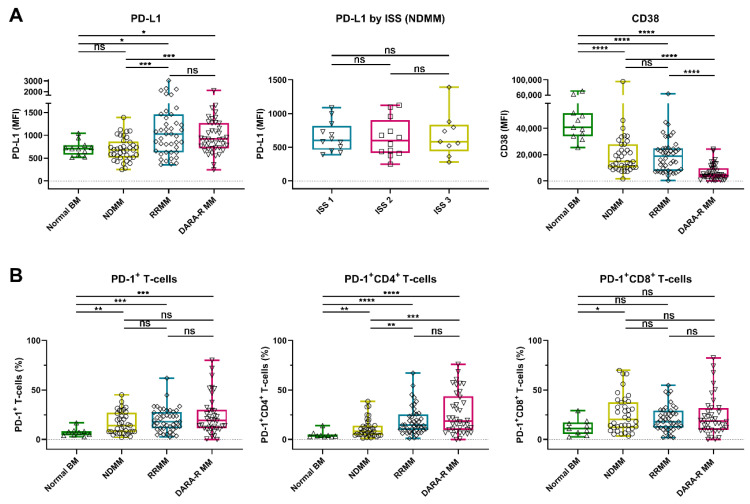
Expression of PD-L1 and CD38 on tumor cells and frequency of PD-1^+^ T-cells in BM samples from healthy controls and MM patients. (**A**) Expression levels (MFI) of PD-L1 and CD38 on normal plasma cells (*n* = 11) and MM cells (NDMM (*n* = 37), daratumumab-naïve RRMM (*n* = 43), and daratumumab-refractory MM (*n* = 41)) are depicted. For newly diagnosed patients, PD-L1 is given per ISS stage. (**B**) The proportions of PD-1^+^ T-cells, PD-1^+^ CD4^+^ T-cells, and PD-1^+^ CD8^+^ T-cells are shown per category (normal BM (*n* = 9), NDMM (*n* = 37), daratumumab-naïve RRMM (*n* = 43), and daratumumab-refractory MM (*n* = 38)). Data are depicted as individual points with box and whiskers, indicating the median, quartiles, and range. Indicated groups were compared using Mann–Whitney tests. Abbreviations: BM, bone marrow; MM, multiple myeloma; MFI, median fluorescence intensity; NDMM, newly diagnosed MM; RRMM, relapsed/refractory MM; ISS, International Staging System; ns, not significant; * *p* ≤ 0.05; ** *p* ≤ 0.01; *** *p* ≤ 0.001; **** *p* ≤ 0.0001.

We detected a marked heterogeneity in the expression levels of PD-L1 on the MM cell surface in these patients’ samples, with the median fluorescence intensity (MFI) ranging from 246 to 6660 (median 808). No difference in PD-L1 expression was observed between normal plasma cells (PCs) from the healthy controls and malignant PCs from NDMM patients (median MFI 709 vs. 680). However, PD-L1 expression was significantly higher on MM cells from daratumumab-naïve RRMM patients (median MFI 1030, *p* = 0.016) and daratumumab-refractory MM patients (median MFI 918, *p* = 0.030), when compared to normal PCs, or when compared to NDMM (*p* < 0.001 for both daratumumab-naïve and daratumumab-refractory RRMM). No significant difference in PD-L1 expression was observed between daratumumab-naïve and daratumumab-refractory patients (Figure 1A and Figure S1A). NDMM patients with International Staging System (ISS) stage 3 disease had similar PD-L1 expression as patients with stage 1 or 2 (Figure 1A).

We also examined the frequency of PD-1^+^ T-cells in the BM of these patients. An increased proportion of PD-1^+^ T-cells was found in BM samples obtained from NDMM (median 14.1%, *p* = 0.009), daratumumab-naïve RRMM (median 17.9%, *p* < 0.001), and daratumumab-refractory MM patients (median 19.0%, *p* < 0.001), when compared to healthy controls (median 7.1%). The frequency of PD-1^+^ CD4^+^ T-cells was higher in in RRMM (daratumumab-naïve and daratumumab-refractory) when compared to NDMM, while this difference was not observed in CD8^+^ T-cells (Figure 1B and Figure S1B).

In the same set of patients’ samples, we also analyzed the CD38 expression on MM cells by using the CD38 antibody HuMax-003, which binds to an epitope distinct from the epitope bound by daratumumab. This enables the analysis of CD38 expression irrespective of ongoing or recent daratumumab treatment. In accordance with our previous studies, in this larger cohort of patients, the CD38 expression levels were similar in patients with NDMM (median MFI 14749) or daratumumab-naïve RRMM (median MFI 19015), while CD38 expression was significantly lower in patients with daratumumab-refractory disease (median MFI 4544, *p* < 0.0001) [10]. Normal PCs had the highest levels of CD38 expression (median MFI 40801, *p* < 0.0001; Figure 1A).

#### 2.1.2. PD1/PD-L1 Axis and CD38 Expression in Primary Plasma Cell Leukemia

Primary plasma cell leukemia (pPCL) is the most aggressive plasma cell disorder. Because of the low incidence of pPCL, there is currently no data available on PD-L1 and PD-1 cell surface expression levels. We therefore analyzed BM samples from 8 patients presenting with pPCL and observed that the expression levels of PD-L1 were similar on tumor cells derived from pPCL or NDMM patients (Figure S2). In addition, there was no difference in the proportion of PD-1^+^ T-cells between both plasma cell dyscrasias, also when the CD4^+^ and CD8^+^ T-cells were analyzed separately. However, CD38 expression was significantly lower on tumor cells from pPCL patients compared to NDMM patients (*p* = 0.037).

### 2.2. Daratumumab-Mediated MM Cell Lysis in Short-Term Ex Vivo Assays

#### 2.2.1. PD-L1 Expression Levels Are Not Associated with the Extent of Daratumumab-Mediated MM Cell Lysis

We and others have demonstrated that daratumumab has immunomodulatory effects, and that daratumumab treatment results in changes in the frequency of PD-1^+^ T-cells [15]. We therefore hypothesized that part of the heterogeneity in response to daratumumab is due to naturally occurring differences in the expression of PD-1/PD-L1. Hence, we first analyzed the impact of the PD-L1 expression levels on the MM cells and on the extent of daratumumab-mediated lysis by using 49 BM samples obtained from daratumumab-naïve patients (including 5 patients with pPCL). The clinical characteristics of these patients are shown in Table 1.

**Figure 2:**
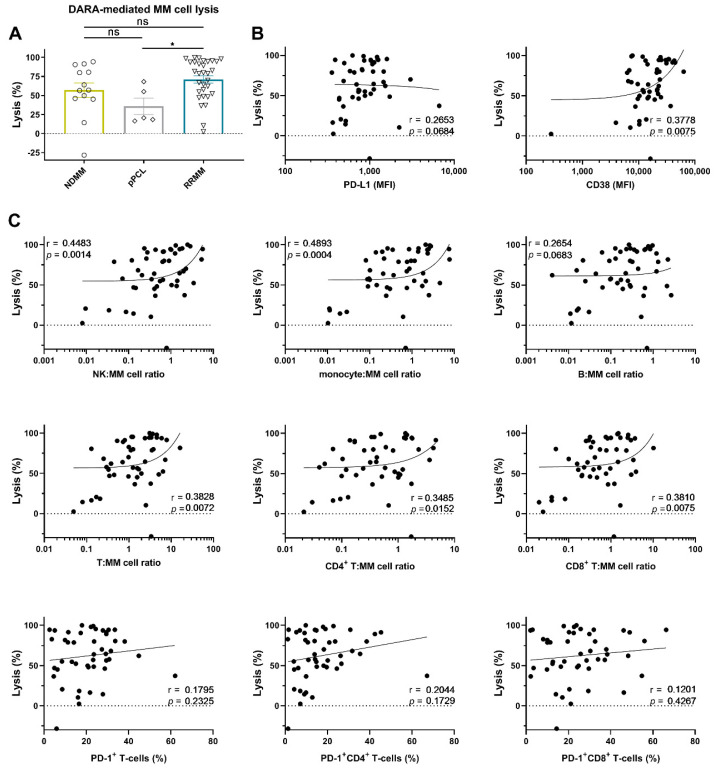
Daratumumab-mediated MM cell lysis in short-term ex vivo cytotoxicity assays. (**A**) BM-MNCs obtained from 13 NDMM, 5 pPCL, and 31 daratumumab-naïve RRMM patients were incubated with daratumumab 10 µg/mL in duplicate for 48 h, after which MM cell-specific lysis was assessed by flow cytometric analysis. Groups were compared using the Mann–Whitney test; data are depicted as individual experiments, with bars representing the mean ± SEM. The correlations between daratumumab-mediated MM cell lysis and (**B**) the baseline tumor characteristics, and (**C**) the immune characteristics, including the effector:target ratios, were calculated using Spearman’s correlation coefficient (r). The median NK-cell:MM cell ratio was 0.64 (range 0.01–5.63); the median monocyte:MM cell ratio was 0.66 (range 0.01–7.70); the median B-cell:MM cell ratio was 0.26 (range 0.00–2.69); the median T-cell:MM cell ratio was 1.32 (range 0.05–16.18); the median CD4^+^ T-cell:MM cell ratio was 0.46 (range 0.02–4.67); the median CD8^+^ T-cell:MM cell ratio was 0.66 (range 0.02–10.21). Dots represent individual experiments. Abbreviations: pPCL, primary plasma cell leukemia; MFI, median fluorescence intensity; ns, not significant; * *p* ≤ 0.05.

To this end, BM mononuclear cells (BM-MNCs), containing tumor cells, autologous effector cells, and immune suppressive cells, were incubated for 48 h with 10 µg/mL daratumumab, after which MM cell survival was determined by enumeration of viable CD138^+^ cells by flow cytometric analysis. Daratumumab-mediated lysis was very heterogeneous and ranged from −28 (negative values indicate an increase in MM cell numbers; daratumumab non-responsive) to 100% (median lysis: 67%). No significant difference in daratumumab-mediated lysis between NDMM and RRMM was observed (Figure 2A). Although the numbers are small, daratumumab-mediated lysis in the pPCL samples was similar to that observed in samples from NDMM patients, but lower when compared to RRMM patients (Figure 2A, *p* = 0.022). Across all BM samples, daratumumab-mediated lysis was not correlated with cell-surface expression levels of PD-L1 in these short-term killing assays. However, in accordance with our previous findings [11], there was a significant positive association between CD38 expression levels on MM cells from patients and the efficacy of daratumumab to induce cell death (r = 0.38; *p* = 0.008) (Figure 2B). Figure S3A shows the correlation between daratumumab-mediated lysis and PD-L1 or CD38 expression, whereby samples from newly diagnosed or daratumumab-naïve RRMM patients were analyzed separately.

#### 2.2.2. Effector (NK-Cell, T-Cell, and Monocyte)-to-Target Ratio is Associated with the Extent of Daratumumab-Mediated MM Cell Lysis

We have previously shown that, in short-term killing assays, daratumumab kills MM cells predominantly via engagement of monocytes and NK-cells [30,31]. However, the impact of T-cells in these short-term experiments is currently unknown. Because the patient-derived BM samples contained variable amounts of immune effector cells, we also examined whether the frequency of different immune cell subsets, including T-cells, in the BM microenvironment contributes to the variability in response to daratumumab.

As expected, both a high monocyte: MM cell ratio (r = 0.49; *p* = 0.0004) and high NK: MM cell ratio (r = 0.45; *p* = 0.0014) were associated with improved daratumumab-mediated lysis of MM cells, while the B-cell:MM cell ratio had no impact on daratumumab activity. The T-cell to MM cell ratio was also positively correlated with the extent of daratumumab-mediated killing of MM cells (r = 0.38; *p* = 0.007, Figure 2C). However, the proportion of PD-1^+^ T-cells was not correlated with the susceptibility of primary MM cells to daratumumab in these short-term assays. Similar observations were made when CD4^+^ and CD8^+^ T-cells were analyzed separately. Figure S3B shows the correlation between daratumumab-mediated lysis and the frequency of the immune cell subsets, whereby samples from newly diagnosed or daratumumab-naïve RRMM patients were analyzed separately.

### 2.3. Combining Nivolumab and Daratumumab in Short-Term In Vitro Experiments

**Figure 3:**
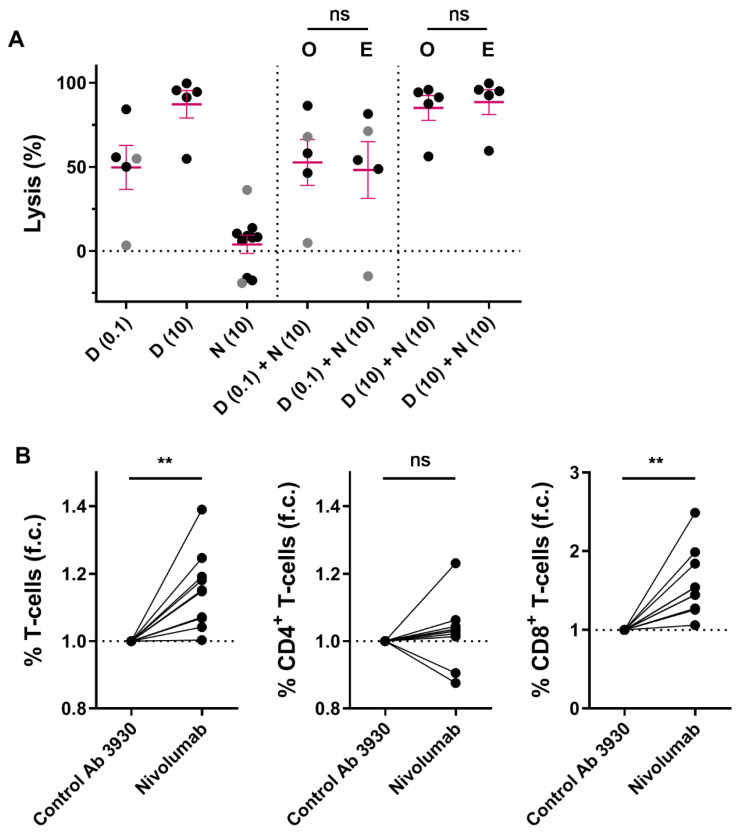
Short-term ex vivo co-treatment with nivolumab does not enhance daratumumab-mediated MM cell lysis. (**A**) BM-MNCs derived from 2 NDMM (grey) and 8 daratumumab-naïve RRMM (black) patients were treated with daratumumab (0.1 µg/mL (*n* = 5) or 10 µg/mL (*n* = 5)), nivolumab 10 µg/mL, or the combination of both antibodies for 48 h, after which MM cell specific lysis was determined by using flow cytometry. Dots represent an individual data point, and error bars represent the mean ± SEM; experiments were performed in duplicate. The observed MM cell lysis in samples treated with both daratumumab and nivolumab was compared with the expected lysis, which was calculated using the Bliss method as described in the Materials and Methods. The *p*-values between observed and expected lysis were calculated by using paired Student’s *t*-tests. (**B**) The fold changes in the CD3^+^, CD4^+^, and CD8^+^ T-cell frequencies were calculated by dividing the % T-cells (of live BM-MNCs) in wells treated with nivolumab (10 µg/mL) by the % T-cells in wells treated with CNTO 3930 control antibody (10 µg/mL) (*n* = 10). Paired Student’s *t*-tests were used to calculate significance. Abbreviations: D, daratumumab; N, nivolumab; SEM, standard error of mean; O, observed lysis; E, expected lysis; ns, not significant; ** *p* ≤ 0.01, f.c., fold change.

We hypothesized that immune modulation through targeting CD38 combined with blockade of the PD-1/PD-L1 pathway may lead to improved T-cell activity, and thereby better anti-MM efficacy. Although no correlations between PD-L1 or PD-1 expression levels and daratumumab-mediated killing were observed, we further investigated whether the combination of daratumumab and nivolumab, a fully human PD-1 blocking antibody, enhances MM cell death in short-term ex vivo cytotoxicity assays. To this end, we treated 10 different BM samples from MM patients with nivolumab (10 µg/mL (*n* = 10)), daratumumab (0.1 µg/mL (*n* = 5), or 10 µg/mL (*n* = 5)), or the combination of both antibodies for 48 h. Although blockade of PD-1 with nivolumab did not noticeably affect MM cell viability or enhance daratumumab-mediated MM cell killing in these short-term ex vivo experiments (Figure 3A), nivolumab treatment resulted in a mild but clear increase in T-cell frequency (1.15 fold increase compared to control antibody; *p* = 0.003; Figure 3B), which is predominantly driven by an increase in CD8^+^ T-cells (1.59 fold increase; *p* = 0.001), while the CD4^+^ T-cells remain unchanged (1.03 fold increase; *p* = 0.43).

We also assessed the effect of nivolumab on daratumumab-mediated MM cell lysis in an assay with longer incubation time (96 h), by using healthy donor-derived peripheral blood mononuclear cells (PB-MNCs) and MM cell lines. To this end, PB-MNCs were first pre-incubated with or without nivolumab (10 µg/mL) for 48 h, and then added to MM.1S or UM9 cells in the presence of control antibody or daratumumab (0.1 or 10 µg/mL), with or without nivolumab (10 µg/mL), for another 48 h. Pre-incubating PB-MNCs with nivolumab did not enhance daratumumab-mediated MM cell lysis (Figure S4).

### 2.4. Combined Targeting of PD-1 and CD38 in Mouse Tumor Models

#### 2.4.1. Mechanisms of Action of the Anti-Mouse CD38 Antibody

**Figure 4:**
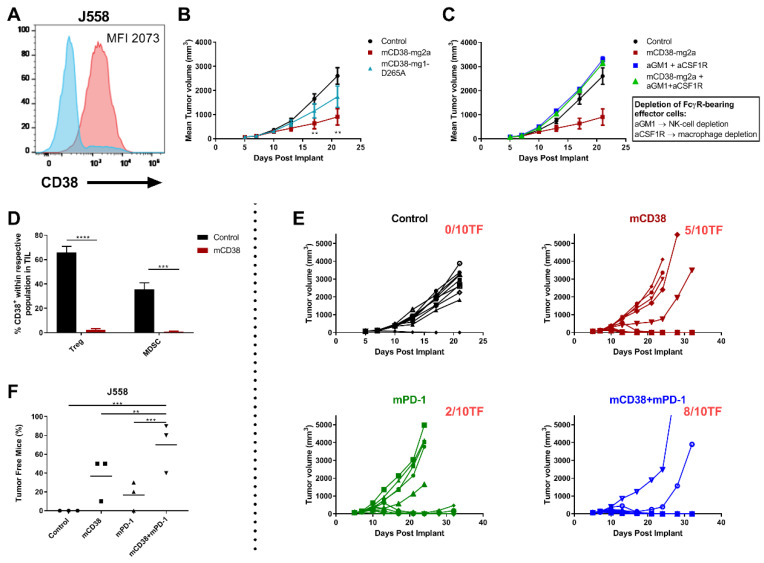
Enhanced antitumor activity with the combination of anti-mCD38 and anti-mPD-1 in a murine myeloma tumor model. (**A**) A representative flow cytometry histogram overlay, depicting CD38 cell surface expression on J558 MM cells (red histogram), compared with the isotype control (blue histogram). The median fluorescence intensity (MFI) is provided. (**B**) Treatment of mice with FcR-engaging or FcγR-inert mAbs. BALB/c mice were injected subcutaneously with J558 tumor cells (5 × 10^6^ cells) and randomized at day 6 when their tumors reached an average size of 90–100 mm^3^. Mice were injected intraperitoneally with 10 mg/kg of either control mIgG2a and control mIgG1-D265A (black), anti-mCD38 mIg2a (red), or anti-mCD38 mIgG1-D265A (inert antibody, blue) on days 6, 10, and 13. Data are the mean ± SEM (*n* = 10 mice/group). (**C**) Effect of depletion of FcγR-bearing effector cells. J558 tumor-bearing mice were injected intraperitoneally with anti-asialo-GM1 (resulting in NK-cell depletion) and anti-CSF1R (resulting in macrophage depletion) on day 5 (1 day prior to anti-mCD38 mAb treatment) and every 7 days thereafter. Data are the mean ± SEM (*n* = 10 mice/group). (**D**) Tumor-infiltrating mononuclear cells from J558 tumor-bearing mice were harvested on day 11 post-implantation, following treatment with anti-mCD38 or mIgG2a control mAb. Percentage of CD38^+^ cells within population of Tregs (%CD4^+^Foxp3^+^) or MDSCs (%Gr1^+^CD11b^+^) is shown. Data are depicted as the mean ± SEM and are from a representative experiment with 5 mice per group. (**E**) BALB/c mice were injected subcutaneously with J558 tumor cells (5 × 10^6^ cells) and randomized at day 6 when their tumors reached an average size of 90–100 mm^3^. Mice were injected intraperitoneally with 10 mg/kg of either control mIgG2a or anti-mCD38 mAb, 0.3 mg/kg anti-mPD-1, or combination of anti-mCD38/anti-mPD-1 mAbs on days 6, 10, and 13. Tumor volumes for individual mice and the number of tumor-free mice per group are shown; *n* = 10 per group. This experiment is representative for a total of 3 independent experiments. (**F**) Fisher’s Exact test (two-sided) analysis to determine between-treatment differences in the proportion of J558-bearing mice that were tumor free. Three independent tumor studies were performed with 10 mice per treatment per study. Symbols represent different studies within a treatment group. Abbreviations: mAb, monoclonal antibody; TIL, tumor-infiltrating lymphocytes; SEM, standard error of mean; ** *p* ≤ 0.01, *** *p* ≤ 0.001, **** *p* ≤ 0.0001.

In these short-term co-culture experiments, daratumumab predominantly kills MM cells via its direct on-tumor mechanisms of action, while the immunomodulatory effects require a longer period of time [12,13,15]. Therefore, we further explored the combined effect of targeting PD-1 and CD38 in the endogenously CD38 expressing mouse myeloma J558 tumor model (Figure 4A). Daratumumab does not bind to mouse CD38, and therefore in these in vivo experiments, an anti-mouse CD38 antibody (anti-mCD38) was used that exhibited characteristics of daratumumab, i.e., mediated in vitro ADCC and ADCP of mouse CD38-expressing tumor cells (Figure S5).

We first evaluated the activity and mechanism of action of anti-mCD38 mAb in the J558 tumor-bearing model. Enhanced anti-tumor activity was observed using the FcγR-engaging anti-mCD38 mIgG2a monoclonal antibody (mAb), compared to the FcγR-inert anti-mCD38 mIgG1-D265A mAb. At day 21 post implantation, J558 tumor-bearing mice that received anti-mCD38 mIgG2a demonstrated a mean tumor volume of 904 ± 339 mm^3^, compared to 1742 ± 442 mm^3^ in the anti-mCD38 mIgG1-D265A group and 2604 ± 334 mm^3^ in the control-treated group (*p* < 0.01) (Figure 4B). Furthermore, the activity of the FcγR-engaging antibody was significantly decreased in the absence of FcγR-bearing macrophages and NK-cells (Figure 4C). Hence, these data indicate that FcγR-engagement of the anti-CD38 antibody is necessary for its optimal antitumor activity in vivo. In addition, similar to what is observed in patients treated with daratumumab [12], treatment of mice with the anti-mCD38 antibody resulted in a significant reduction of tumor-infiltrating CD38^+^ Tregs (%CD4^+^Foxp3^+^: 2.5 ± 0.8% vs. 65.8 ± 10.9% in the control group, *p* < 0.0001) and CD38^+^ MDSCs (%Gr1^+^CD11b^+^: 3.7 ± 1.2% vs. 42.9 ± 2.7% in the control group, *p* < 0.001) (Figure 4D).

#### 2.4.2. Enhanced Anti-Tumor Activity with the Combination of Anti-mCD38 and Anti-mPD-1 in a Murine Myeloma Tumor Model

Next, we evaluated the combined effect of targeting PD-1 and CD38 in J558 tumor-bearing mice. To this end, 6 days after the injection of 5 × 10^6^ J558 tumor cells, mice were randomized to treatment with a mIgG2a control antibody, anti-mPD-1 monotherapy, anti-mCD38 monotherapy, or the combination of both therapeutic antibodies (*n* = 10 mice/group; 3 independent experiments). Overall, the anti-mCD38/anti-mPD-1 combination treatment resulted in a significant increase in the number of tumor-free mice (70%), compared with mice treated with the control antibody (0%, *p* < 0.001), single-agent anti-mCD38 (37%, *p* = 0.004), or anti-mPD-1 (17%, *p* < 0.001) (Figure 4F). Figure 4E shows a representative example of one of these three independent experiments, showing superior anti-tumor activity of the anti-mCD38/anti-mPD-1 combination, compared to either agent alone.

#### 2.4.3. Enhanced Antitumor Activity with the Combination of Anti-mCD38 and Anti-mPD-1 in the CD38-Expressing MC38 Tumor Model

**Figure 5:**
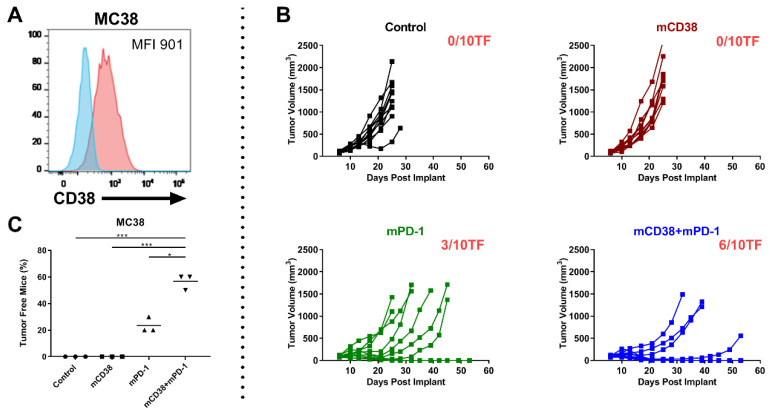
Combination of anti-mCD38 and anti-mPD-1 show enhanced antitumor activity in a CD38-expressing MC38 tumor model. (**A**) A representative flow cytometry histogram overlay, depicting CD38 cell surface expression on MC38 colon carcinoma cells (red histogram), compared with the isotype control (blue histogram). The median fluorescence intensity (MFI) is provided. (**B**) C57BL/6 mice were injected subcutaneously with MC38 tumor cells (1 × 10^6^ cells) and randomized at day 6 when their tumors reached an average size of 80–100 mm^3^. Mice were injected intraperitoneally with 10 mg/kg of either control mIgG2a, anti-mCD38 mAb, anti-mPD-1, or combination of anti-mCD38/anti-mPD-1 mAbs on days 6, 10, 13, 17, and 20. Tumor volumes for individual mice and the number of tumor-free mice per group are shown; *n* = 10 per group. This experiment is representative of a total of three independent experiments. (**C**) Fisher’s Exact test (two-sided) was used to determine the between-treatment differences in the proportion of MC38-bearing mice that were tumor free. Three independent tumor studies were performed with 10 mice per treatment per study. Symbols represent different studies within a treatment group. Abbreviations: * *p* ≤ 0.05, *** *p* ≤ 0.001.

To analyze whether the anti-mCD38/anti-mPD-1 combination is also effective in other CD38^+^ tumors, we tested the combination of anti-mPD-1 and anti-mCD38 treatment in mice with established MC38 colon adenocarcinoma tumors, which also express CD38 (Figure 5A). Six days after the injection of 5 × 10^6^ MC38 tumor cells, mice were randomized to treatment with a mIgG2a control antibody, anti-mPD-1 monotherapy, anti-mCD38 monotherapy, or the combination of both therapeutic antibodies (*n* = 10 mice/group; 3 independent experiments). Treatment of mice with both anti-mPD1 and anti-mCD38 resulted in a significantly increased proportion of tumor-free mice (57%), compared to control-treated mice (0%, *p* < 0.001) or mice treated with anti-mPD-1 alone (24%, *p* = 0.017) or anti-mCD38 alone (0%, *p* < 0.001) (Figure 5C). A representative example of one of these three independent experiments is shown in Figure 5B. Taken together, the combination of anti-mCD38 and anti-mPD-1 antibodies showed superior efficacy to either agent alone in mouse tumor models that express CD38 on tumor cells (both J558 and MC38), even when no single-agent antitumor activity was observed.

## 3. Discussion

MM exploits multiple mechanisms to avoid the host’s immune response, which, together with genetic aberrations, likely contribute to tumor progression. A better understanding of such mechanisms underlying immune evasion by MM cells may result in better treatment strategies.

In our analysis of fresh BM samples obtained from MM patients, we show similar levels of PD-L1 on plasma cells from NDMM patients, but significantly increased PD-L1 expression levels in patients with RRMM, compared to normal plasma cells from healthy controls of comparable age. There was no further increase in PD-L1 expression in heavily pretreated daratumumab-refractory MM patients with six prior lines of therapy, compared to RRMM patients with less extensive pretreatment. We also demonstrate a significantly increased proportion of PD-1^+^ T-cells in both NDMM and RRMM patients, compared to healthy controls. A similar expression of PD-1 and PD-L1 was observed in NDMM and pPCL, the most aggressive plasma cell malignancy. Overall, our results are consistent with several other studies [22,23,24,25,32], and expand our knowledge on the PD-1/PD-L1 axis in heavily pretreated MM and pPCL. Altogether, this indicates that there is increased expression of the PD-1/PD-L1 pathway components in the BM microenvironment during MM progression, which may contribute to the gradual attenuation of T-cell mediated immune responses during the patient’s disease course [21].

Daratumumab has pleiotropic mechanisms of action, including immunomodulatory effects by elimination of CD38^+^ immune suppressor cells, resulting in increased T-cell numbers and T-cell killing capacity [12,13]. Based on these findings, we hypothesized that daratumumab may be an appropriate candidate for combination therapy with other T-cell based immunotherapies, such as the PD-1 blocking antibody nivolumab. Since the PD-1/PD-L1 interaction has been shown to inhibit T-cell mediated lysis and T-cell proliferation [28,33,34], we first performed short-term ex vivo assays with nivolumab in the presence or absence of daratumumab. In these assays, nivolumab moderately increased the CD8^+^ T-cell numbers, but had no anti-MM activity nor enhanced the anti-tumor activity of daratumumab. We also observed no correlation between daratumumab-mediated MM cell death and PD-1 expression on T-cells or PD-L1 expression on MM cells. Indeed, in these short-term experiments, daratumumab predominantly kills tumor cells via direct on-tumor mechanisms of action (ADCC and ADCP), which require the presence of CD38 on the tumor cell surface and Fc-receptor-bearing immune effector cells (i.e., NK-cells and macrophages) in the BM microenvironment [10,11]. This is in accordance with our findings showing that high CD38 expression levels on MM cells and high effector-to-target ratios contribute to more efficient daratumumab-mediated MM cell lysis. In contrast, the immunomodulatory effects of daratumumab, including the increase in T-cell numbers and enhanced T-cell activity, require a longer period of time [12,13,15]. We therefore hypothesized that improved efficacy of daratumumab through PD-1/PD-L1 blockade may be uncovered in in vivo models. Indeed, combined targeting of CD38 and PD-1 demonstrated enhanced antitumor activity in a MM mouse tumor model. For this purpose, we used syngeneic tumor models in immunocompetent mice, and we were able to validate several clinical observations in these mice, including the daratumumab-mediated elimination of CD38^+^ Tregs and CD38^+^ MDSCs [12,13,15]. Altogether our data indicate that MM patients may benefit from the combination of antibodies targeting PD-1/PD-L1 and CD38, which both activate the immune system against MM cells, but through different mechanisms of action. Although the evaluation of checkpoint inhibitors in combination with IMiDs in MM has been halted because of an unfavorable benefit/risk profile of the PD-1 inhibitor pembrolizumab plus either lenalidomide-dexamethasone or pomalidomide-dexamethasone [35,36], several trials evaluating PD-1/PD-L1 blockade (e.g., nivolumab or atezolizumab) combined with daratumumab are ongoing in patients with RRMM (NCT03184194, NCT01592370, NCT02431208). A limitation of our study is that we did not assess daratumumab-mediated CDC in the in vitro experiments. However, the mice used in the in vivo studies have exceptionally low complement activity, compared to humans [37]. This suggests that daratumumab-mediated CDC is, most likely, not the main driver for the enhanced anti-tumor activity when anti-CD38 and anti-PD-1 antibodies are combined.

Blockade of the PD-1/PD-L1 pathway is effective in patients with several types of solid tumors. However, some tumor types do not respond to PD-1/PD-L1 blockade and a lack of durable response has been observed in patients with responsive tumor types. There is increasing evidence that CD38 antibodies, next to eliminating CD38^+^ immune suppressor cells [12], may have additional immunomodulatory effects. Indeed, a recent study showed that CD38 is upregulated on lung cancer cells following blockade of the PD-1/PD-L1 pathway [38]. The enhanced CD38 expression resulted in impaired T-cell function, through induction of increased adenosine (an immune suppressive molecule) production via its ectoenzymatic activity [39,40]. Importantly, CD38 antibodies inhibit CD38 ectoenzymatic activity [38,41] and rapidly reduce CD38 expression on tumor cells as well as non-tumor immune cells [10,30]. Taken together, these mechanisms may contribute to reduced CD38-mediated adenosine production, an improved host anti-tumor immune response, and the observed synergy between PD-1 blockade and a CD38-targeting antibody in the murine colon cancer model. These findings are in concordance with those of Chen et al., who recently reported that co-blockade of CD38 and PD-1 improves the antitumor immune response in lung cancer mouse models [38].

In conclusion, our in vivo data show that anti-CD38 and anti-PD-1 antibodies synergize to eradicate CD38-expressing tumor cells. Based on our preclinical findings, this combined immunotherapeutic approach is currently evaluated in several clinical trials with RRMM patients. In addition, our results suggest that this combination may also have a role in the treatment of other malignancies, where targeting CD38 may enhance the efficacy of PD-1/PD-L1 checkpoint inhibitors.

## 4. Materials and Methods

### 4.1. Patients

Cell surface expression of PD-L1 was assessed in 129 BM aspirates, obtained from 37 newly diagnosed (ND) MM patients, 8 primary plasma cell leukemia patients (pPCL), 43 daratumumab-naïve relapsed/refractory (RR) MM patients, and 41 daratumumab-refractory MM patients. Normal BM (*n* = 11) was obtained from patients undergoing cardiothoracic surgery, with a comparable age as the MM patients. In these samples, we concurrently evaluated several tumor characteristics and the immune cell composition. Ex vivo efficacy of daratumumab was assessed when BM samples contained at least 2% MM cells and sufficient material was available. The samples were analyzed within 24 h after BM aspiration.

The study site ethics committee (METc VUmc, study registration number 2017.159/NL60544.029.17) or institutional review board approved the protocols, which were conducted according to the principles of the Declaration of Helsinki, the International Conference on Harmonization, and the Guidelines for Good Clinical Practice. All patients provided written informed consent.

### 4.2. Antibodies and Reagents

Daratumumab was provided by Janssen Pharmaceuticals (Beerse, Belgium) and nivolumab by Bristol-Myers Squibb (New York City, NY, USA). The nonspecific human IgG1 antibody CNTO 3930 (Janssen Pharmaceuticals) was used as the isotype control.

The following monoclonal antibodies (mAbs) were used in mouse (m) tumor models: anti-mCD38-FcγR-engaging mAb (mIgG2a isotype; this antibody mediates ADCC/ADCP of mCD38^+^ tumor cells); anti-mCD38 FcγR-inert mAb (mIgG1-D265A isotype; this antibody has no FcγR-mediated effector function); and anti-mPD-1 FcγR-inert mAb (mIgG1-D265A isotype; blocks mPD-1:mPD-L1/mPD-L2 interactions [42,43]). Control antibodies included mIgG2a (clone C1.18.4, BioXCell, Lebanon, NH, USA) and mIgG1-D265A (Bristol-Myers Squibb).

### 4.3. Bone Marrow Mononuclear Cells

BM mononuclear cells (BM-MNCs) from BM aspirates obtained from healthy donors and MM patients were isolated using Ficoll–Hypaque density-gradient centrifugation within 24 h of sampling.

### 4.4. Flow Cytometric Analysis of BM Samples from MM Patients and Healthy Controls

BM-localized normal and malignant plasma cells were identified and analyzed for cell surface marker expression levels by staining 1.0 × 10^6^ cells/mL with CD138 PE, CD56 PC7, CD45 Krome Orange (all Beckman Coulter, Brea, CA, USA), CD274 (PD-L1) BV421, and CD19 APC-H7 (both BD Biosciences, San Jose, CA, USA). Cells were also stained with HuMax-003 FITC (Genmab, Copenhagen, Denmark/Janssen Pharmaceuticals), which binds to a CD38 epitope distinct from the epitope bound by daratumumab. This enables the analysis of CD38 expression in BM samples irrespective of ongoing or recent daratumumab treatment [30].

Immune cell subsets in whole BM aspirates were identified and analyzed for cell surface marker expression levels by staining 1.0 × 10^6^ cells/mL with CD45 Krome Orange, CD56 PC7 (both Beckman Coulter), CD14 APC-H7, CD19 APC-H7, CD3 V450, CD4 APC-H7 or PE, CD8 FITC, CD279 (PD-1) BV421, CD16 APC (all BD Biosciences), and CD38 HuMax-003 FITC.

Flow cytometry was performed using a 7-laser LSRFORTESSA (BD Biosciences). Fluorescent-labeled beads (CS & T beads, BD Biosciences) were used daily to monitor the performance of the flow cytometer and verify optical path and stream flow. This procedure enables controlled standardized results and allows the determination of long-term drifts and incidental changes within the flow cytometer. No changes were observed that could affect the results. Compensation beads were used to determine the spectral overlap, and compensation was automatically calculated using FACSDiva software (BD Biosciences). Flow cytometry data were analyzed using FCS Express Flow software (Version 6, De Novo Software, Pasadena, CA, USA).

### 4.5. Flow Cytometry-Based Ex Vivo Cytotoxicity Assays in BM-MNCs

BM-MNCs derived from multiple myeloma (MM) patients, containing 2–67% CD138^+^ tumor cells, as well as autologous effector cells and immune suppressive cells, were used in the flow cytometry-based lysis assays. Sample viability at start of the assays, assessed using 7-AAD (BD Biosciences), was more than 95%. BM-MNCs were incubated in RPMI + 10% Fetal Bovine Serum with human IgG1 control antibody or daratumumab (0.1–10 µg/mL) in 96-well U-bottom plates for 48 h. For combination treatment assays, cells were also incubated with or without nivolumab (10 µg/mL). The survival of primary CD138^+^ MM cells was determined by flow cytometry, as previously described [10,11,44,45]. Briefly, surviving MM cells were enumerated by single platform flow cytometric analysis of CD138^+^ cells in the presence of Flow-Count Fluorospheres (Beckman Coulter) and LIVE/DEAD Fixable Dead Cell Stain Near-IR fluorescent reactive dye (Invitrogen, Carlsbad, CA, USA). The percentage of lysis induced by daratumumab and/or nivolumab was calculated using the following formula, as described previously [11]:

% lysis MM cells= 1− (absolute number of surviving MM cells in treated wells)/(absolute number of surviving MM cells in untreated wells) × 100.

### 4.6. Bioluminescence Imaging-Based Cytotoxicity Assays Using Human MM Cell Lines

The luciferase (LUC)-transduced MM cell lines UM9 and MM.1S were cultured in RPMI-1640 (Invitrogen, Carlsbad, CA, USA), supplemented with 10% HyClone FetalClone I serum (GE Healthcare Life Sciences, Marlborough, MA, USA) and antibiotics (100 units/mL penicillin, 100 μg/mL streptomycin). UM9 was obtained after prolonged in vitro culture of the BM aspirate of a MM patient. MM.1S was purchased from the American Tissue Culture Collection (ATCC, Manassas, VA, USA). Monthly mycoplasma testing was performed using real-time PCR (Microbiome). Cell lines were authenticated by short-tandem repeat profiling carried out maximal 6 months before the most recent experiment. Cell lines were used for a time period no longer than 4 months.

PB-MNCs were first pre-incubated with or without nivolumab (10 µg/mL) for 48 h, and then added to the MM cell lines in the presence of daratumumab (0.1–10 µg/mL) or the control antibody with or without nivolumab (10 µg/mL) for another 48 h. The survival of the LUC^+^-MM cells was determined by bioluminescence imaging (BLI), 30 min after addition of the substrate luciferin (150 µg/mL; Promega, Madison, WI, USA). MM cell lysis was calculated using the following formula:

% lysis = 1 − (mean BLI signal in treated wells) /(mean BLI signal in untreated wells) × 100.

### 4.7. In Vitro ADCC and ADCP Assays

For the antibody-dependent cellular cytotoxicity (ADCC) assays, human NK-cells were isolated from healthy donor PB-MNCs by negative selection using a magnetic bead-based separation kit (StemCell Technologies, Vancouver, BC, Canada) and cultured in MyeloCult media (StemCell Technologies) with 500 IU/mL human recombinant IL-2 for 24 h. NK-cells were cocultured in a 96-well V-bottom plate with J558 target cells (mouse MM cell line) labeled with calcein at a 10:1 ratio, in the presence of anti-mCD38 hIgG1 or hIgG1 control mAb. After a 2-h incubation at 37 °C, specific lysis in the supernatant was measured by the fluorescence (485 nm excitation and 515 nm emission) using a Perkin Elmer Envision device. Percentage of specific lysis was calculated using the formula:

Percentage of Specific Lysis= (Experimental Release Medium Release)/(Maximum Release Medium Release) × 100.

For antibody-dependent cellular phagocytosis (ADCP) assays, U937 effector cells were cocultured with J558 target cells labeled with PKH26 (Millipore Sigma, Burlington, MA, USA) at a 1:4 effector-to-target ratio in the presence of a fixed concentration of control hIgG1 mAb or increasing doses of anti-mCD38 hIgG1 mAb. After a 1-h incubation at 37 °C, cells were washed and stained with APC labeled anti-CD89 mAb (Biolegend, San Diego, CA, USA) and percent phagocytosis (% PKH26 among CD89^+^ cells) was measured by flow cytometry.

### 4.8. Mice

C57BL/6 or BALB/c female mice were purchased from Charles River (Indianapolis, IN, USA) or Envigo (Frederick, MD, USA). All mice were housed at Bristol-Myers Squibb (Princeton, NJ, USA) and the mouse experimentation was carried out subsequent to proper review and approval by the Bristol-Myers Squibb Animal Care and Use Committee, accredited by the Association for Assessment and Accreditation of Laboratory Animal Care International (accreditation #001085). All mice were maintained under specific pathogen-free conditions following the “Guide for the Care and Use of Laboratory Animals” and used between 6 to 12 weeks of age.

### 4.9. Mouse Cell Lines and Culturing Conditions

MC38, a mouse colon adenocarcinoma cell line endogenously expressing mCD38, and J558, a mouse myeloma line endogenously expressing mCD38 (ATCC, Manassas, VA, USA), were maintained in Dulbecco’s modified Eagle medium (DMEM) with 10% fetal bovine serum (FBS).

### 4.10. Tumor Cell Implantation and Treatment

J558-derived tumors were established via subcutaneous injection of 5 × 10^6^ J558 cells into the hind flanks of Balb/c mice. After 6 days, when the tumor volumes reached 90 to 100 mm^3^, mice were randomized into treatment groups. In experiments addressing mechanisms of action of the anti-mouse CD38 antibody, mice were injected intraperitoneally with 10 mg/kg of either anti-mCD38 mAb, an isotype control antibody, or an anti-mCD38 FcγR-inert mAb on days 6, 10, and 13. To study the effect of depletion of the FcγR-bearing effector cells, mice were treated with anti-asialo-GM1 (resulting in NK-cell depletion) and anti-CSF1R (resulting in macrophage depletion) on day 5 (1 day prior to anti-mCD38 mAb treatment) and every 7 days thereafter. In the J558 combinatorial treatment model, anti-mCD38 and anti-mPD-1 mAbs were dosed at 10 mg/kg and 0.3 mg/kg, respectively. The mAbs were administered intraperitoneally at days 6, 10, and 13 post implantation for a total of 3 doses.

The MC38 tumors were established via subcutaneous injection of 1 × 10^6^ MC38 cells into the hind flank of C57BL/6 mice. After 6 days, when the tumor volumes reached 80 to 100 mm^3^, mice were randomized into treatment groups. Antibodies were administered intraperitoneally at days 6, 10, 13, 17, and 20 post implantation for a total of 5 doses. In the MC38 model, anti-mCD38 and anti-mPD-1 mAbs were dosed at 10 mg/kg. Tumor growth was determined biweekly by using electronic digital calipers (Fowler, Newton, MA, USA). Mice with a tumor volume of 0 mm^3^ for 3 consecutive measurements were considered tumor free.

### 4.11. Immunophenotypic Analysis

J558 tumors were resected on day 11 post implantation. Single-cell suspensions were prepared by dissociation and passing cells through a 70-µm filter. Cells (1 × 10^6^) were plated in 96-well plates, treated with Fc block (clone 2.4G2; BD Biosciences), and stained with fluorochrome-conjugated antibodies against surface or intracellular markers (Table S1). Expression of mCD38 among tumor-infiltrating immune subsets was performed using a fluorochrome-conjugated mouse CD38 antibody that was determined to bind a different epitope than the anti-mCD38 treatment antibody. For intracellular staining, samples were fixed, permeabilized, and stained with antibodies (FoxP3 staining buffer set; eBioscience, San Diego, CA, USA). Antibody fluorescence was detected by flow cytometry on Fortessa (BD Biosciences), and the results were analyzed using FlowJo^TM^ software (FlowJo, Ashland, OR, USA).

### 4.12. Statistics

Comparisons between variables were performed using two-tailed (paired) Student’s *t*-tests or Mann–Whitney U tests in case the data did not follow a normal distribution. Spearman’s rank correlation was used to assess the correlation between different variables, in case the data did not follow a normal distribution. In case of combinatorial treatment with nivolumab and daratumumab, the expected lysis values were calculated, using the following formula that assumes that there is an additive effect between the combined agents (Bliss model):

% expected lysis = [(% lysis with nivolumab) + (% lysis with daratumumab)] – [(% lysis with nivolumab) × (% lysis with daratumumab)],

as described before [31,45,46]. Flow cytometry data from the mouse experiments were analyzed by unpaired Student’s *t*-tests. Differences in the proportion of tumor-free mice in the isotype control, anti-mCD38, anti-mPD-1, and anti-mCD38/mPD-1 treatment groups were determined using Fisher’s exact test (two-sided). *p*-values below 0.05 were considered significant. Statistical analyses were performed in GraphPad Prism (version 8, San Diego, CA, USA).

## 5. Conclusions

In this study, we demonstrate that during MM progression there is increased expression of the PD-1/PD-L1 pathway components in the bone marrow microenvironment. In short-term ex vivo experiments with bone marrow samples from MM patients, daratumumab-mediated lysis was mainly associated with the CD38 expression levels on MM cells and the effector-to-target ratio, but not with PD-L1 expression levels or proportion of PD-1^+^ T-cells.

Although PD-1 blockade with nivolumab did not affect MM cell viability or enhance daratumumab-mediated lysis in short-term ex vivo experiments, nivolumab resulted in a mild but clear increase in T-cell numbers. Moreover, with a longer treatment duration, PD-1 blockade markedly improved anti-CD38 antibody-mediated cytotoxicity in vivo in murine CD38^+^ tumor models. In conclusion, dual targeting of CD38 and PD-1 may represent a promising strategy for treating MM and other CD38-positive malignancies.

## Figures and Tables

**Table 1:** Patient characteristics.

Characteristic		NDMM (*n* = 13)	pPCL (*n* = 5)	RRMM (*n* = 31)
Age, median (range)		75 (31–86)	65 (57–97)	66 (46–77)
Male sex, *n* (%)		7 (54)	2 (40)	16 (52)
M protein, *n* (%)				
	IgG	6 (50)	2 (40)	22 (71)
	IgA	0	0	4 (13)
	FLC only	6 (50)	3 (60)	5 (16)
	Unknown	1 (8)	0	0
ISS score, *n* (%)				
	1	2 (29)	0	n.a.
	2	3 (43)	1 (25)	
	3	2 (29)	3 (75)	
	Unknown	6 (46)	1 (20)	
High risk cytogenetics ^a^, *n* (%)		1 (8)	3 (75)	6 (23)
	Unknown	1 (8)	1 (20)	5 (16)
	Del (17p)	1 (8)	3 (75)	4 (15)
	t (4;14)	1 (8)	1 (25)	2 (8)
	t (14;16)	0	0	0
Prior lines of therapy, median (range)		0	0	3 (1–9)
Prior autologous SCT, *n* (%)				
	Single	n.a.	n.a.	17 (61)
	Double			2 (7)
Prior allogeneic SCT, *n* (%)		n.a.	n.a.	2 (7)
Lenalidomide, *n* (%)				
	Exposed	n.a.	n.a.	27 (87)
	Refractory ^b^			26 (84)
Pomalidomide, *n* (%)				
	Exposed	n.a.	n.a.	16 (52)
	Refractory ^b^			16 (52)
Bortezomib, *n* (%)				
	Exposed	n.a.	n.a.	27 (87)
	Refractory			13 (42)
Carfilzomib, *n* (%)				
	Exposed	n.a.	n.a.	9 (29)
	Refractory			7 (23)
Daratumumab, *n* (%)				
	Exposed	n.a.	n.a.	0
	Refractory			0
IMiD and PI ^c^				
	Exposed	n.a.	n.a.	27 (87)
	Refractory			18 (58)

^a^ Based on the presence of del (17p), t (4;14), and/or t (14;16), some patients have more than one abnormality. ^b^ Refractory disease was defined as progressive disease during therapy, no response (less than PR), or progressive disease within 60 days of stopping treatment, according to the International Uniform Response Criteria for Multiple Myeloma. ^c^ Lenalidomide and/or pomalidomide plus bortezomib and/or carfilzomib. Abbreviations: HD, healthy donor; NDMM, newly diagnosed MM; RRMM, relapsed/refractory MM; DARA-R MM, daratumumab refractory MM; FLC, free light chains; ISS, International Staging System; SCT, stem cell transplantation; IMiD, immunomodulatory drug; PI, proteasome inhibitor; n.a., not applicable.

## Supplementary Materials

The following are available online at https://www.mdpi.com/2072-6694/12/12/3713/s1, Table S1: Antibodies used in mouse experiments, Figure S1: Flow cytometry histogram plots of PD-L1 expression on plasma cells and dot plots of PD-1 expression on T-cells, Figure S2: Expression of PD-L1, CD38 and PD-1 in pPCL and NDMM samples, Figure S3: Correlation between daratumumab-mediated MM cell lysis and baseline tumor and immune characteristics in samples derived from newly diagnosed or relapsed/refractory patients, Figure S4: Pre-treatment of peripheral blood mononuclear cells (PB-MNCs) with nivolumab does not enhance short-term in vitro daratumumab-mediated lysis of MM cell lines, Figure S5: Anti-mCD38 mAb mediates ADCC and ADCP in vitro.
