# Supplementary material for: Preclinical Rationale for Targeting the PD-1/PD-L1 Axis in Combination with a CD38 Antibody in Multiple Myeloma and Other CD38-Positive Malignancies
Source: Cancers (Basel). 2020 Dec 10;12(12):3713. doi: 10.3390/cancers12123713 (PMC7764511; doi:10.3390/cancers12123713)
Supplement: Supplementary file 1 [file cancers-12-03713-s001.pdf]

Supplementary Materials: Preclinical Rationale for Targeting the PD-1/PD-L1 Axis in Combination with a CD38 Antibody in Multiple Myeloma and Other CD38-Positive Malignancies

Christie P.M. Verkleij, Amy Jhatakia, Marloes E.C. Broekmans, Kristine A. Frerichs, Sonja Zweegman, Tuna Mutis, Natalie A. Bezman, Niels W.C.J. van de Donk

Table S1. Antibodies used in mouse experiments.

Mouse Antibodies			
**Antigen**	**Conjugate**	**Manufacturer**	**Antibody Clone/Catalog Number**
CD3	PE	eBioscience/Thermo Fisher Scientific (Waltham, MA, USA)	2C11
CD8	AF700	BioLegend	53-6.7
CD11b	BV421	Biolegend	M1/70
CD38	AF488	BioLegend	90
CD45	BV510	BioLegend	30-F11
Foxp3	PE-Cy7	eBioscience	FJK-16s
GR1	APC	Biolegend	RB6-8C5
LIVE/DEAD dye	Near-IR	Thermo Fisher Scientific	Catalog# L10119
Ly-6C	FITC	Biolegend	HK1.4

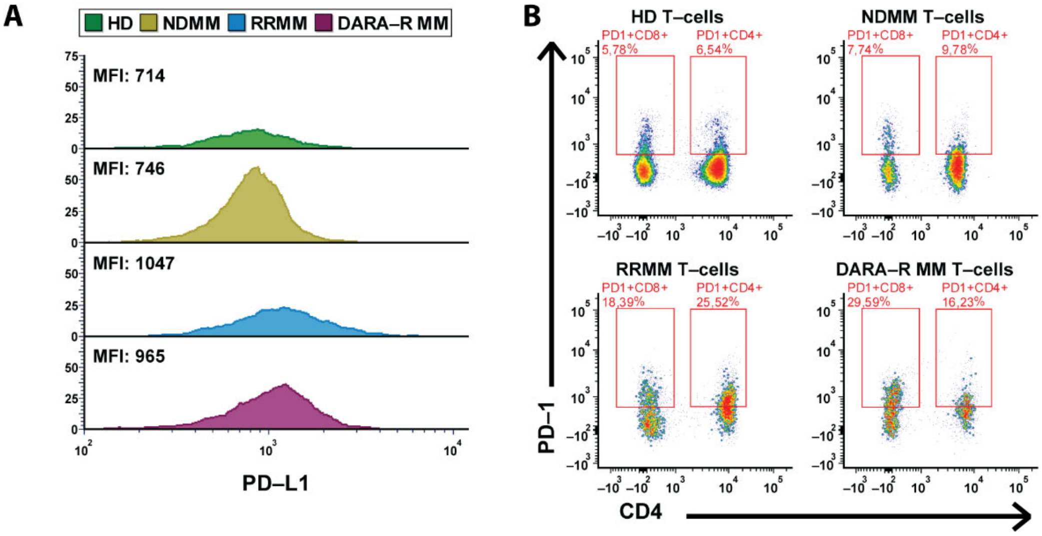

Figure S1. Flow cytometry histogram plots of PD-L1 expression on plasma cells and dot plots of PD-1 expression on T-cells. (**A**) Representative flow cytometry histogram plots depicting PD-L1 cell surface expression on normal plasma cells from a healthy donor (HD, green) and on malignant plasma cells obtained from a newly diagnosed (NDMM, yellow), relapsed/refractory (RRMM, blue) and daratumumab-refractory patient (DARA-R MM, purple). Median Fluorescence Intensity (MFI) values are included in the plots. (**B**) Representative flow cytometry dot plots showing the proportion of PD-1^{+} (*y*-axis) CD4^{+} and CD8^{+} T-cells in bone marrow samples obtained from a healthy donor (HD) or from multiple myeloma (MM) patients in different stages of their disease (NDMM, RRMM and DARA-R MM). The cut-off for PD-1 positivity is based on fluorescence intensity of cells not stained with PD-1. Percentages of PD-1^{+} T-cells in these samples are shown.

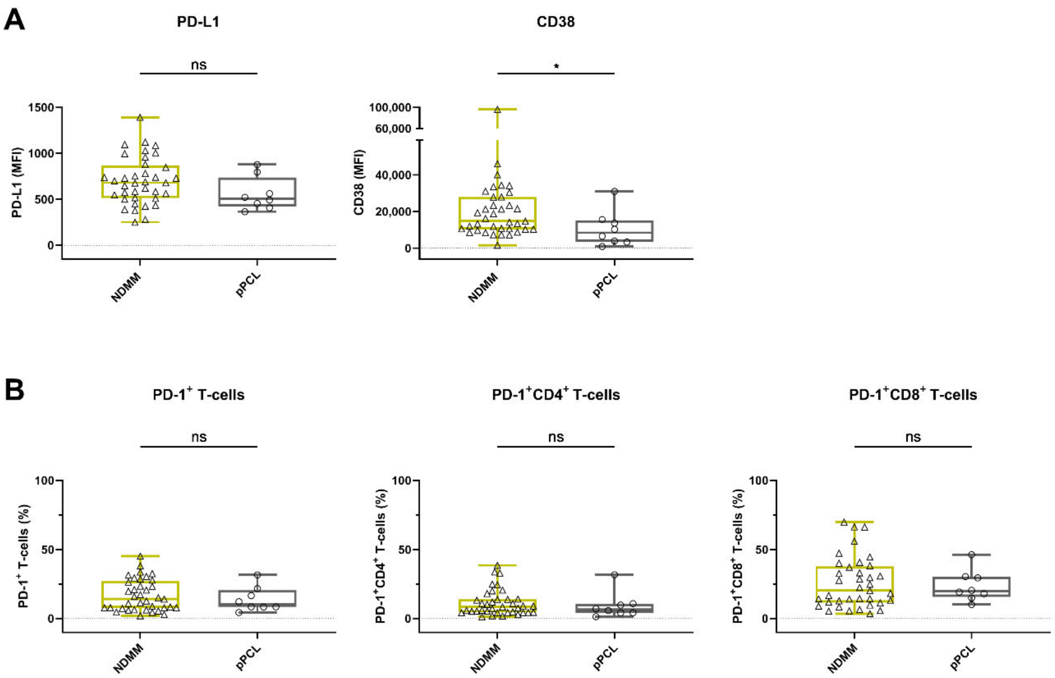

Figure S2. Expression of PD-L1, CD38 and PD-1 in pPCL and NDMM samples. (**A**) Expression levels of PD-L1 and CD38 on tumor cells from NDMM (n = 37) or primary plasma cell leukemia patients (pPCL, n = 8). (**B**) Proportion of PD-1$^+$ T-cells in bone marrow samples derived from NDMM (n = 37) and pPCL (n = 8) patients. Expression levels were assessed by flow cytometry. Data are depicted as individual points with box and whiskers, indicating median, quartiles and range. Groups were compared using Mann-Whitney test. Abbreviations: MFI, median fluorescence intensity; ns, not significant; * $p \leq 0.05$.

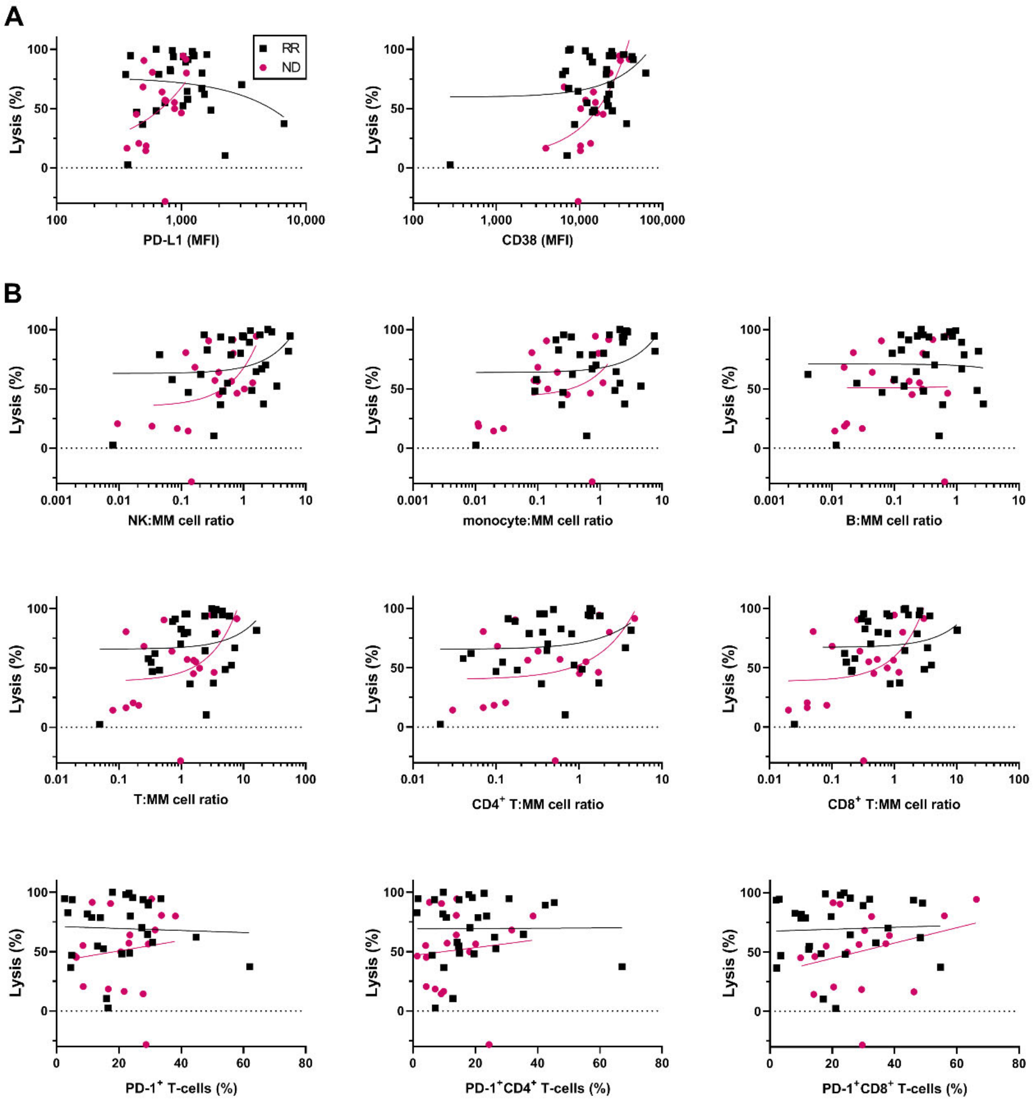

		PD-L1	CD38	NK:MM	mono:MM	B:MM	T:MM	$CD4^+$ T :MM	$CD8^+$ T :MM	$PD\text{-}1^+$ T	$PD\text{-}1^+$ $CD4^+$ T	$PD\text{-}1^+$ $CD8^+$ T
ND	Spearman r	0.4324	0.7503	0.4634	0.4283	0.2405	0.4306	0.4202	0.4760	0.3251	0.2549	0.3581
	p-value	0.0731	0.0003	0.0528	0.0762	0.3365	0.0745	0.0825	0.0459	0.1881	0.3073	0.1445
RR	Spearman r	0.0087	0.2077	0.3709	0.3909	0.1337	0.2961	0.3442	0.2454	0.0892	0.0443	0.0985
	p-value	0.9637	0.2623	0.0436	0.0327	0.4812	0.1121	0.0626	0.1912	0.6516	0.8227	0.6179

Figure S3. Correlation between daratumumab-mediated MM cell lysis and baseline tumor and immune characteristics in samples derived from newly diagnosed or relapsed/refractory patients. BM-MNCs obtained from 18 newly diagnosed (ND) patients and 31 daratumumab-naïve relapsed/refractory (RR) patients were incubated with daratumumab 10 µg/mL in duplicate for 48 hours, after which MM cell specific lysis was assessed by flow cytometric analysis. The correlation between daratumumab-mediated MM cell lysis and specified (**A**) baseline tumor characteristics and (**B**) immune characteristics, including effector:target ratios, was calculated using Spearman's correlation coefficient (r), whereby samples from newly diagnosed or relapsed/refractory patients were analyzed separately. Red dots represent individual experiments with samples from ND patients, and black dots represent individual experiments with samples from RR patients. Correlation coefficients and *p*-values are presented in the table, significant values are blue. Abbreviations: BM-MNCs, bone marrow mononuclear cells; MFI, median fluorescence intensity.

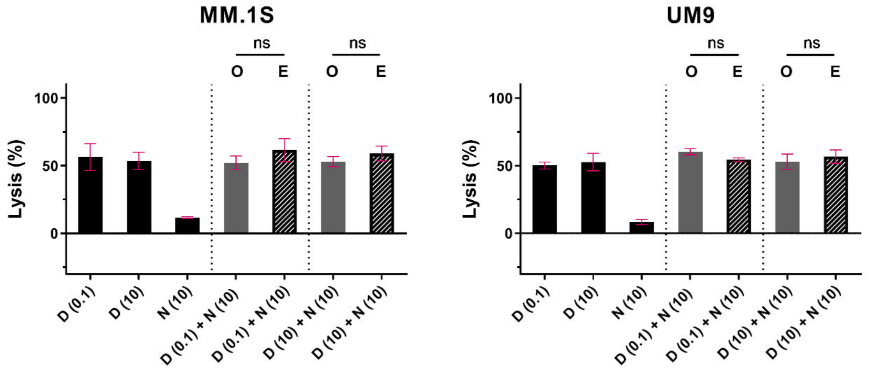

Figure S4. Pre-treatment of peripheral blood mononuclear cells (PB-MNCs) with nivolumab does not enhance short-term in vitro daratumumab-mediated lysis of MM cell lines. PBMCs were first pre-incubated for 48 hours with or without nivolumab (10 µg/mL), and then added to luciferase-transduced MM.1S or UM9 cell lines in the presence of daratumumab (0.1 or 10 µg/mL), with or without nivolumab (10 µg/mL), for another 48 hours. MM cell lysis was determined using bioluminescence imaging. Individual experiments were performed in triplicate (n = 2), error bars represent mean ± SEM. The observed MM cell lysis in wells treated with both daratumumab and nivolumab (grey bars) was compared with the expected lysis (striped bars), which was calculated using the Bliss method as described in the Materials and Methods. *p*-values between observed and expected lysis were calculated using a paired Student *t*-test. Abbreviations: D, daratumumab; N, nivolumab; ns, not significant; SEM, standard error of mean.

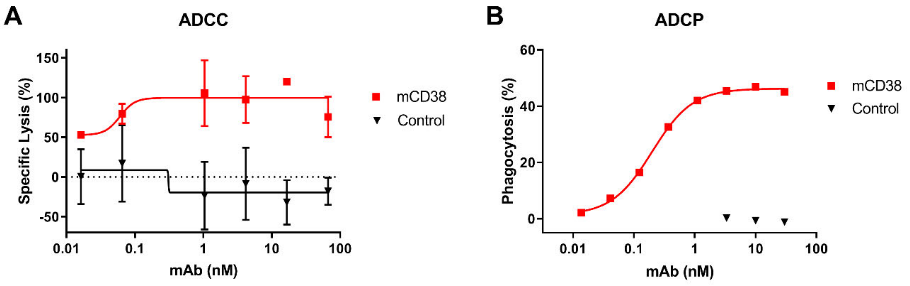

Figure S5. Anti-mCD38 mAb mediates ADCC and ADCP in vitro. (**A**) NK-cells from healthy donors were incubated with calcein labeled J558 cells at a 10:1 ratio for 2 hours of either increasing doses of control hIgG1 or anti-mCD38 (hIgG1) mAb. The percent of specific lysis is shown. Results are representative of 4 donors. (**B**) U937 effector cells were co-cultured with PKH26-labeled J558 target cells at a 1:4 ratio in the presence of control hIgG1 or increasing doses of anti-mCD38 (hIgG1) mAb. The percent of phagocytosis (% PKH26 among $CD89^{+}$ cells) is shown. Abbreviations: ADCC, antibody-dependent cellular cytotoxicity; ADCP, antibody-dependent cellular phagocytosis; mAB, monoclonal antibody.

Publisher's Note: MDPI stays neutral with regard to jurisdictional claims in published maps and institutional affiliations.

© 2020 by the authors. Licensee MDPI, Basel, Switzerland. This article is an open access article distributed under the terms and conditions of the Creative Commons Attribution (CC BY) license (http://creativecommons.org/licenses/by/4.0/).
